# Supplementary material for: Minocycline as Treatment for Psychiatric and Neurological Conditions: A Systematic Review and Meta-Analysis
Source: Int J Mol Sci. 2023 Mar 9;24(6):5250. doi: 10.3390/ijms24065250 (PMC10049047; doi:10.3390/ijms24065250)

Supplementary materials:

Table S1: PsycINfO search strategy:

SEARCH	QUERY
**#1**	SU Minocycline
**#2**	TI Minocycline
**#3**	AB Minocycline
**#4**	S1 OR S2 OR S3
**#5**	DE (Amphetamine OR Amyotrophic Lateral Sclerosis OR Opiates OR Anxiety OR Benzodiazepines OR Traumatic Brain Injury OR Cannabis OR Cerebrovascular disorders OR Autism Spectrum Disorders OR Cocaine OR Dementia OR Epilepsy OR Headache OR Heroin OR Ischemia OR Kleptomania OR Mental Disorders OR Methamphetamine OR Methylphenidate OR Migraine Headache OR Multiple Sclerosis OR Nail Biting OR Neurodegenerative Diseases OR Neurology OR Neuroethology OR Neuropathology OR Nicotine OR Psychiatry OR Seizures OR Substance Use Disorder)
**#6**	TI "neurological disorder" OR neurology OR autism OR "autistic disorder" OR ASD OR Asperger's OR "Asperger Syndrome" OR "pervasive developmental disorder" OR "Alzheimer’s disease" OR dementia OR "amyotrophic lateral sclerosis" OR ALS OR seizures OR epilepsy OR "traumatic brain injury" OR TB OR stroke OR ischemia OR haemorrhage OR neuropathy OR "peripheral neuropathy" OR neurodegenerative OR "Parkinson disease" OR "Huntington disease" OR "brain injury" OR "multiple sclerosis" OR "spinal cord injury" OR migraine OR headache OR "psychiatric disorder" OR "mental illness" OR "mental disorders" OR anxiety OR addiction OR "major depressive disorder" OR "major depression" OR "depressive disorder" OR MDD OR "bipolar I disorder" OR "bipolar II disorder" OR "bipolar disorder" OR "bipolar depression" OR mania OR "manic disorder" OR "manic state" OR hypo-mania OR psychosis OR "psychotic disorders" OR schizophrenia OR "schizoaffective disorder" OR "panic disorder" OR "social anxiety disorder" OR "posttraumatic stress disorder" OR "generalised anxiety disorder" OR OCD OR "personality disorder" OR "obsessive compulsive disorder" OR "obsessive-compulsive neurosis" OR "obsessive-compulsive neuroses" OR "attention deficit hyperactivity disorder" OR ADHD OR trauma OR "stress disorders" OR "post traumatic" OR "feeding disorder" OR "appetite disorder" OR disruptive, impulse control, conduct disorders OR "eating disorder" OR "binge eating disorder" OR "bulimia nervosa" OR "anorexia nervosa" OR "other specified feeding and eating disorder" OR methylphenidate OR amphetamine OR methamphetamine OR cocaine OR cannabis OR marijuana OR heroin OR "prescription pills" OR opioids OR benzodiazepine OR nicotine OR tobacco OR "pathological gambling" OR "skin picking" OR "impulse control disorder" OR kleptomania
**#7**	AB "neurological disorder" OR neurology OR autism OR "autistic disorder" OR ASD OR Asperger's OR "Asperger Syndrome" OR "pervasive developmental disorder" OR "Alzheimer’s disease" OR dementia OR "amyotrophic lateral sclerosis" OR ALS OR seizures OR epilepsy OR "traumatic brain injury" OR TB OR stroke OR ischemia OR haemorrhage OR neuropathy OR "peripheral neuropathy" OR neurodegenerative OR "Parkinson disease" OR "Huntington disease" OR "brain injury" OR "multiple sclerosis" OR "spinal cord injury" OR migraine OR

	headache OR "psychiatric disorder" OR "mental illness" OR "mental disorders" OR anxiety OR addiction OR "major depressive disorder" OR "major depression" OR "depressive disorder" OR MDD OR "bipolar I disorder" OR "bipolar II disorder" OR "bipolar disorder" OR "bipolar depression" OR mania OR "manic disorder" OR "manic state" OR hypo-mania OR psychosis OR "psychotic disorders" OR schizophrenia OR "schizoaffective disorder" OR "panic disorder" OR "social anxiety disorder" OR "posttraumatic stress disorder" OR "generalised anxiety disorder" OR OCD OR "personality disorder" OR "obsessive compulsive disorder" OR "obsessive-compulsive neurosis" OR "obsessive-compulsive neuroses" OR "attention deficit hyperactivity disorder" OR ADHD OR trauma OR "stress disorders" OR "post traumatic" OR "feeding disorder" OR "appetite disorder" OR disruptive, impulse control, conduct disorders OR "eating disorder" OR "binge eating disorder" OR "bulimia nervosa" OR "anorexia nervosa" OR "other specified feeding and eating disorder" OR methylphenidate OR amphetamine OR methamphetamine OR cocaine OR cannabis OR marijuana OR heroin OR "prescription pills" OR opioids OR benzodiazepine OR nicotine OR tobacco OR "pathological gambling" OR "skin picking" OR "impulse control disorder" OR kleptomania
**#8**	S5 OR S6 OR S7
**#9**	(DE "Treatment Effectiveness Evaluation") OR (DE "Treatment Outcomes" OR DE "Psychotherapeutic Outcomes" OR DE "Side Effects (Treatment)" OR DE "Treatment Compliance" OR DE "Treatment Duration" OR DE "Treatment Refusal" OR DE "Treatment Termination" OR DE "Treatment Withholding") OR (DE "Placebo") OR (DE "Followup Studies") OR placebo* OR random* OR "comparative stud*" OR (clinical N3 trial*) OR (research N3 design) OR (evaluat* N3 stud*) OR (prospectiv* N3 stud*) OR ((singl* OR doubl* OR trebl* OR tripl*) N3 (blind* OR mask*))
**#10**	S4 AND S8 AND S9
**#11**	S4 AND S8 AND S9 Limiters: English; Human

Table S2: Embase search strategy:

SEARCH	QUERY
**#1**	minocycline:ab,ti
**#2**	minocycline/de
**#3**	#1 OR #2
**#4**	'amphetamine'/exp OR 'amyotrophic lateral sclerosis'/exp OR 'opiate'/exp OR 'anxiety'/exp OR 'benzodiazepine derivative'/exp OR 'brain injury'/exp OR 'cannabis'/exp OR 'cerebrovascular disease'/exp OR 'autism'/exp OR 'cocaine'/exp OR 'dementia'/exp OR 'headache and facial pain'/exp OR 'diamorphine'/exp OR 'ischemia'/exp OR 'impulse control disorder'/exp OR 'mental disease'/exp OR 'methamphetamine'/exp OR 'methylphenidate'/exp OR 'multiple sclerosis'/exp OR 'degenerative disease'/exp OR 'neuropathology'/exp OR 'neurology'/exp OR 'nicotine'/exp OR 'psychiatry'/exp OR 'seizure, epilepsy and convulsion'/exp OR 'drug dependence'/exp
**#5**	'neurological disorder':ab,ti OR neurology:ab,ti OR autism:ab,ti OR 'autistic disorder':ab,ti OR asd:ab,ti OR aspergers:ab,ti OR 'asperger syndrome':ab,ti OR 'pervasive developmental disorder':ab,ti OR 'alzheimers disease':ab,ti OR dementia:ab,ti OR 'amyotrophic lateral sclerosis':ab,ti OR als:ab,ti OR seizures:ab,ti OR epilepsy:ab,ti OR 'traumatic brain injury':ab,ti OR tb:ab,ti OR stroke:ab,ti OR ischaemia:ab,ti OR haemorrhage:ab,ti OR neuropathy:ab,ti OR 'peripheral neuropathy':ab,ti OR neurodegenerative:ab,ti OR 'parkinson disease':ab,ti OR 'huntington disease':ab,ti OR 'brain injury':ab,ti OR 'multiple sclerosis':ab,ti OR 'spinal cord injury':ab,ti OR migraine:ab,ti OR headache:ab,ti OR 'psychiatric disorder':ab,ti OR 'mental illness':ab,ti OR 'mental disorders':ab,ti OR anxiety:ab,ti OR addiction:ab,ti OR 'major depressive disorder':ab,ti OR 'major depression':ab,ti OR 'depressive disorder':ab,ti OR mdd:ab,ti OR 'bipolar i disorder':ab,ti OR 'bipolar ii disorder':ab,ti OR 'bipolar disorder':ab,ti OR 'bipolar depression':ab,ti OR mania:ab,ti OR 'manic disorder':ab,ti OR 'manic state':ab,ti OR 'hypo mania':ab,ti OR psychosis:ab,ti OR 'psychotic disorders':ab,ti OR schizophrenia:ab,ti OR 'schizoaffective disorder':ab,ti OR 'panic disorder':ab,ti OR 'social anxiety disorder':ab,ti OR 'posttraumatic stress disorder':ab,ti OR 'generalised anxiety disorder':ab,ti OR ocd:ab,ti OR 'personality disorder':ab,ti OR 'obsessive compulsive disorder':ab,ti OR 'obsessive-compulsive neurosis':ab,ti OR 'obsessive-compulsive neuroses':ab,ti OR 'attention deficit hyperactivity disorder':ab,ti OR adhd:ab,ti OR trauma:ab,ti OR 'stress disorders':ab,ti OR 'post traumatic':ab,ti OR 'feeding disorder':ab,ti OR 'appetite disorder':ab,ti OR 'disruptive, impulse control, conduct disorders':ab,ti OR 'eating disorder':ab,ti OR 'binge eating disorder':ab,ti OR 'bulimia nervosa':ab,ti OR 'anorexia nervosa':ab,ti OR 'other specified feeding and eating disorder':ab,ti OR methylphenidate:ab,ti OR amphetamine:ab,ti OR methamphetamine:ab,ti OR cocaine:ab,ti OR cannabis:ab,ti OR marijuana:ab,ti OR heroin:ab,ti OR 'prescription pills':ab,ti OR opioids:ab,ti OR benzodiazepine:ab,ti OR nicotine:ab,ti OR tobacco:ab,ti OR 'pathological gambling':ab,ti OR 'skin picking':ab,ti OR 'impulse control disorder':ab,ti OR kleptomania:ab,ti
**#6**	#4 OR #5
**#7**	'crossover procedure':de OR 'double-blind procedure':de OR 'randomized controlled trial':de OR 'single-blind procedure':de OR random*:de,ab,ti OR

	factorial*:de,ab,ti OR crossover*:de,ab,ti OR ((cross NEXT/1 over*):de,ab,ti) OR placebo*:de,ab,ti OR ((doubl* NEAR/1 blind*):de,ab,ti) OR ((singl* NEAR/1 blind*):de,ab,ti) OR assign*:de,ab,ti OR allocat*:de,ab,ti OR volunteer*:de,ab,ti
**#8**	#3 AND #6 AND #7 AND [english]/lim
**#9**	#8 AND ([animal cell]/lim OR [animal experiment]/lim OR [animal model]/lim OR [animal tissue]/lim)
**#10**	#8 NOT #9

Table S3. Cochrane search strategy

SEARCH	QUERY
**#1**	(minocycline):kw
**#2**	(minocycline):ab OR (minocycline):ti
**#3**	#1 OR #2
**#4**	(neurology OR Child Development Disorders, Pervasive OR dementia OR amyotrophic lateral sclerosis OR multiple sclerosis OR neurodegenerative diseases OR migraine disorders OR headache disorders OR brain injury, traumatic OR cerebrovascular disorders OR epilepsy OR seizures OR Ischemia OR psychiatry OR mental disorders OR<br>anxiety OR 'Disruptive, Impulse Control, and Conduct Disorders' OR nail biting OR<br>cannabis OR benzodiazepines OR nicotine OR analgesics, opioid OR cocaine OR heroin OR methamphetamine OR amphetamine OR methylphenidate OR (substance-related disorders)):kw
**#5**	("neurological disorder" OR neurology OR autism OR "autistic disorder" OR ASD OR Asperger's OR "Asperger Syndrome" OR "pervasive developmental disorder" OR "Alzheimer's disease" OR dementia OR "amyotrophic lateral sclerosis" OR ALS OR seizures OR epilepsy OR "traumatic brain injury" OR TB OR stroke OR ischaemia OR haemorrhage OR neuropathy OR "peripheral neuropathy" OR neurodegenerative OR "Parkinson disease" OR "Huntington disease" OR "brain injury" OR "multiple sclerosis" OR "spinal cord injury" OR migraine OR headache OR "psychiatric disorder" OR "mental illness" OR "mental disorders" OR anxiety OR addiction OR "major depressive disorder" OR "major depression" OR "depressive disorder" OR MDD OR "bipolar I disorder" OR "bipolar II disorder" OR "bipolar disorder" OR "bipolar depression" OR mania OR "manic disorder" OR "manic state" OR hypo-mania OR psychosis OR "psychotic disorders" OR schizophrenia OR "schizoaffective disorder" OR "panic disorder" OR "social anxiety disorder" OR "posttraumatic stress disorder" OR "generalised anxiety disorder" OR OCD OR "personality disorder" OR "obsessive compulsive disorder" OR "obsessive-compulsive neurosis" OR "obsessive-compulsive neuroses" OR "attention deficit hyperactivity disorder" OR ADHD OR trauma OR "stress disorders" OR "post traumatic" OR "feeding disorder" OR "appetite disorder" OR disruptive, impulse control, conduct disorders OR "eating disorder" OR "binge eating disorder" OR "bulimia nervosa" OR "anorexia nervosa" OR "other specified feeding and eating disorder" OR methylphenidate OR amphetamine OR methamphetamine OR cocaine OR cannabis OR marijuana OR heroin OR "prescription pills" OR opioids OR benzodiazepine OR nicotine OR tobacco OR "pathological gambling" OR "skin picking" OR "impulse control disorder" OR kleptomania):ab OR ("neurological disorder" OR neurology OR autism OR "autistic disorder" OR ASD OR Asperger's OR "Asperger Syndrome" OR "pervasive developmental disorder" OR "Alzheimer's disease" OR dementia OR "amyotrophic lateral sclerosis" OR ALS OR seizures OR epilepsy OR "traumatic brain injury" OR TB OR stroke OR ischaemia OR haemorrhage OR neuropathy OR "peripheral neuropathy" OR neurodegenerative OR "Parkinson disease" OR "Huntington disease" OR "brain injury" OR "multiple sclerosis" OR "spinal cord injury" OR migraine OR headache OR "psychiatric disorder" OR "mental illness"

	OR "mental disorders" OR anxiety OR addiction OR "major depressive disorder" OR "major depression" OR "depressive disorder" OR MDD OR "bipolar I disorder" OR "bipolar II disorder" OR "bipolar disorder" OR "bipolar depression" OR mania OR "manic disorder" OR "manic state" OR hypo-mania OR psychosis OR "psychotic disorders" OR schizophrenia OR "schizoaffective disorder" OR "panic disorder" OR "social anxiety disorder" OR "posttraumatic stress disorder" OR "generalised anxiety disorder" OR OCD OR "personality disorder" OR "obsessive compulsive disorder" OR "obsessive-compulsive neurosis" OR "obsessive-compulsive neuroses" OR "attention deficit hyperactivity disorder" OR ADHD OR trauma OR "stress disorders" OR "post traumatic" OR "feeding disorder" OR "appetite disorder" OR disruptive, impulse control, conduct disorders OR "eating disorder" OR "binge eating disorder" OR "bulimia nervosa" OR "anorexia nervosa" OR "other specified feeding and eating disorder" OR methylphenidate OR amphetamine OR methamphetamine OR cocaine OR cannabis OR marijuana OR heroin OR "prescription pills" OR opioids OR benzodiazepine OR nicotine OR tobacco OR "pathological gambling" OR "skin picking" OR "impulse control disorder" OR kleptomania):ti
**#6**	#4 OR #5
**#7**	#7 #3 AND #6

Table S4: CINAHL search strategy

SEARCH	QUERY
**#1**	MH Minocycline
**#2**	TI Minocycline
**#3**	AB Minocycline
**#4**	S1 OR S2 OR S3
**#5**	(MH "Amphetamine+") OR (MH "Amyotrophic Lateral Sclerosis") OR (MH "Analgesics, Opioid+") OR (MH "Anxiety+") OR (MH "Antianxiety Agents, Benzodiazepine+") OR (MH "Brain Injuries+") OR (MH "Cannabis+") OR (MH "Cerebrovascular Disorders+") OR (MH "Child Development Disorders, Pervasive+") OR (MH "Cocaine+") OR (MH "Dementia+") OR (MH "Epilepsy+") OR (MH "Headache+") OR (MH "Heroin") OR (MH "Ischemia+") OR (MH "Impulse Control Disorders+") OR (MH "Mental Disorders+") OR (MH "Methamphetamine+") OR (MH "Methylphenidate") OR (MH "Multiple Sclerosis+") OR (MH "Neurodegenerative Diseases+") OR (MH "Neurology") OR (MH "Nicotine") OR (MH "Psychiatry+") OR (MH "Seizures+") OR (MH "Substance Use Disorders+")
**#6**	TI "neurological disorder" OR neurology OR autism OR "autistic disorder" OR ASD OR Asperger's OR "Asperger Syndrome" OR "pervasive developmental disorder" OR "Alzheimer's disease" OR dementia OR "amyotrophic lateral sclerosis" OR ALS OR seizures OR epilepsy OR "traumatic brain injury" OR TB OR stroke OR ischemia OR haemorrhage OR neuropathy OR "peripheral neuropathy" OR neurodegenerative OR "Parkinson disease" OR "Huntington disease" OR "brain injury" OR "multiple sclerosis" OR "spinal cord injury" OR migraine OR headache OR "psychiatric disorder" OR "mental illness" OR "mental disorders" OR anxiety OR addiction OR "major depressive disorder" OR "major depression" OR "depressive disorder" OR MDD OR "bipolar I disorder" OR "bipolar II disorder" OR "bipolar disorder" OR "bipolar depression" OR mania OR "manic disorder" OR "manic state" OR hypo-mania OR psychosis OR "psychotic disorders" OR schizophrenia OR "schizoaffective disorder" OR "panic disorder" OR "social anxiety disorder" OR "posttraumatic stress disorder" OR "generalised anxiety disorder" OR OCD OR "personality disorder" OR "obsessive compulsive disorder" OR "obsessive-compulsive neurosis" OR "obsessive-compulsive neuroses" OR "attention deficit hyperactivity disorder" OR ADHD OR trauma OR "stress disorders" OR "post traumatic" OR "feeding disorder" OR "appetite disorder" OR disruptive, impulse control, conduct disorders OR "eating disorder" OR "binge eating disorder" OR "bulimia nervosa" OR "anorexia nervosa" OR "other specified feeding and eating disorder" OR methylphenidate OR amphetamine OR methamphetamine OR cocaine OR cannabis OR marijuana OR heroin OR "prescription pills" OR opioids OR benzodiazepine OR nicotine OR tobacco OR "pathological gambling" OR "skin picking" OR "impulse control disorder" OR kleptomania
**#7**	AB "neurological disorder" OR neurology OR autism OR "autistic disorder" OR ASD OR Asperger's OR "Asperger Syndrome" OR "pervasive developmental disorder" OR "Alzheimer's disease" OR dementia OR "amyotrophic lateral sclerosis" OR ALS OR seizures OR epilepsy OR "traumatic brain injury" OR TB OR

	stroke OR ischemia OR haemorrhage OR neuropathy OR "peripheral neuropathy" OR neurodegenerative OR "Parkinson disease" OR "Huntington disease" OR "brain injury" OR "multiple sclerosis" OR "spinal cord injury" OR migraine OR headache OR "psychiatric disorder" OR "mental illness" OR "mental disorders" OR anxiety OR addiction OR "major depressive disorder" OR "major depression" OR "depressive disorder" OR MDD OR "bipolar I disorder" OR "bipolar II disorder" OR "bipolar disorder" OR "bipolar depression" OR mania OR "manic disorder" OR "manic state" OR hypo-mania OR psychosis OR "psychotic disorders" OR schizophrenia OR "schizoaffective disorder" OR "panic disorder" OR "social anxiety disorder" OR "posttraumatic stress disorder" OR "generalised anxiety disorder" OR OCD OR "personality disorder" OR "obsessive compulsive disorder" OR "obsessive-compulsive neurosis" OR "obsessive-compulsive neuroses" OR "attention deficit hyperactivity disorder" OR ADHD OR trauma OR "stress disorders" OR "post traumatic" OR "feeding disorder" OR "appetite disorder" OR disruptive, impulse control, conduct disorders OR "eating disorder" OR "binge eating disorder" OR "bulimia nervosa" OR "anorexia nervosa" OR "other specified feeding and eating disorder" OR methylphenidate OR amphetamine OR methamphetamine OR cocaine OR cannabis OR marijuana OR heroin OR "prescription pills" OR opioids OR benzodiazepine OR nicotine OR tobacco OR "pathological gambling" OR "skin picking" OR "impulse control disorder" OR kleptomania
**#8**	S5 OR S6 OR S7
**#9**	S9 MH randomized controlled trials (108,104)<br>S10 MH double-blind studies (48,466)<br>S11 MH single-blind studies (14,244)<br>S12 MH random assignment (63,341)<br>S13 MH pretest-posttest design (43,637)<br>S14 MH cluster sample (4,514)<br>S15 TI (randomised OR randomized) (106,915)<br>S16 AB (random*) (315,646)<br>S17 TI (trial) (109,333)<br>S18 MH (sample size) AND AB (assigned OR allocated OR control) (4,054)<br>S19 MH (placebos) (12,846)<br>S20 PT (randomized controlled trial) (122,055)<br>S21 AB (CONTROL W5 GROUP) (112,573)<br>S22 MH (CROSSOVER DESIGN) OR MH (COMPARATIVE STUDIES) (351,031)<br>S23 AB (CLUSTER W3 RCT) (359)<br>S24 MH ANIMALS+ (94,184)<br>S25 MH HUMAN (2,243,062)<br>S26 S24 NOT S25 (85,376)<br>S27 MH (ANIMAL STUDIES) NOT S25 (109,064)<br>S28 TI (ANIMAL MODEL*) NOT MH (HUMAN) (2,959)<br>S29 S26 OR S27 OR S28 (186,848)<br>S30 S9 OR S10 OR S11 OR S12 OR S13 OR S14 OR S15 OR S16 OR S17 OR S18 OR S19 OR S20 OR S21 OR S22 OR S23 (785,019)<br>S31 S30 NOT S29 (747,078)
**#13**	S4 AND S8 AND S31

**#14**	S4 AND S8 AND S31 Limiters: English

Table S5: Publication biases checks

	Rank correlation test for Funnel plot asymmetry			Egger's test	
	Kendall's τ	p			
Schizophrenia				z	p
Total PANSS	-0.273	0.283		-1.352	0.176
Positive PANSS	-0.422	0.108		-1.745	0.081
Negative PANSS	-0.319	0.127		-1.321	0.187
General PANSS	-0.286	0.399		-2.295	0.022
Depressive symptoms	-0.667	0.333		-0.843	0.399
CGI	-0.467	0.272		-2.59	0.01
GAF	-0.667	0.333		-2.246	0.025
Executive Function	-0.4	0.483		-0.805	0.421
Problem Solving	-0.333	0.75		-1.058	0.29
Processing Speed	-0.6	0.233		-2.505	0.012
Verbal Learning	-0.4	0.483		-1.365	0.172
Working Memory	-0.333	0.469		-1.661	0.097
Stroke					
NIHSS	-0.2	0.719		0.407	0.684
mRS	1	0.333		2.049	0.04
Bartel	-0.333	1		-0.166	0.868
mRS category change	-0.333	0.75		0.501	0.617

Figure S1: Funnel Plots

Funnel Plots Schizophrenia

PANSS Total Symptoms:

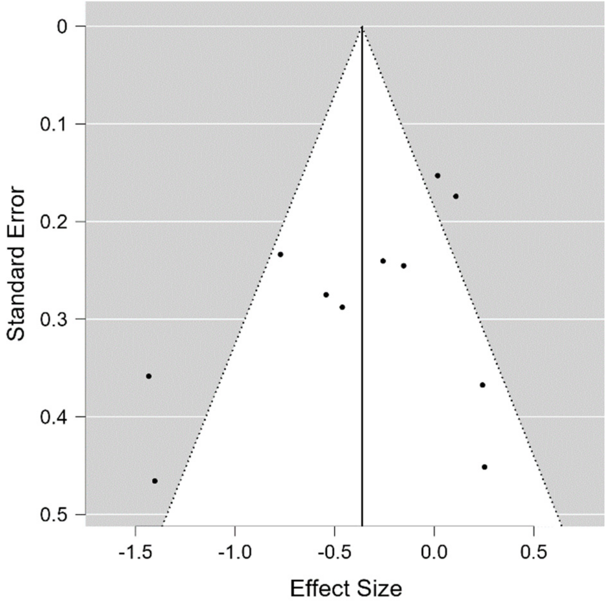

PANSS Negative:

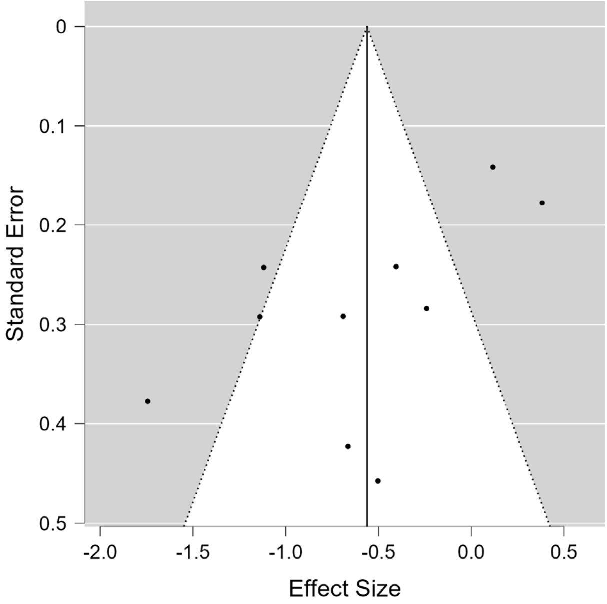

PANSS Positive:

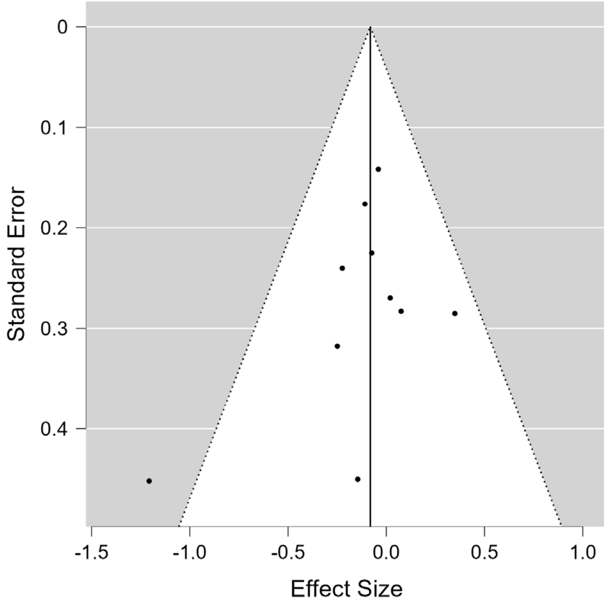

PANSS General

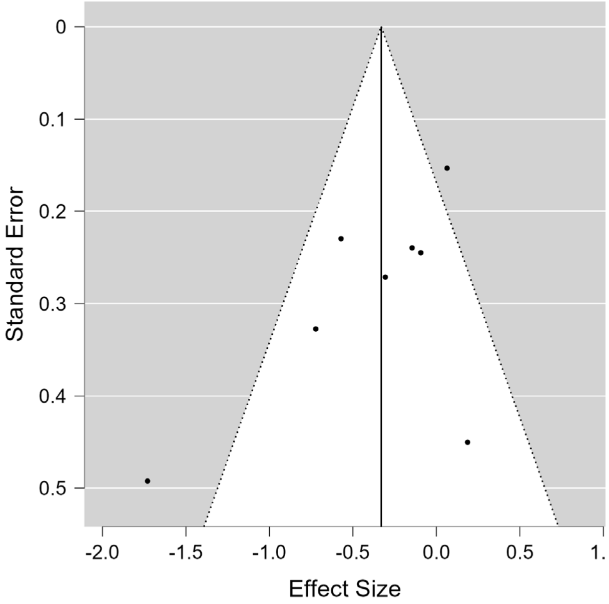

SANS Total:

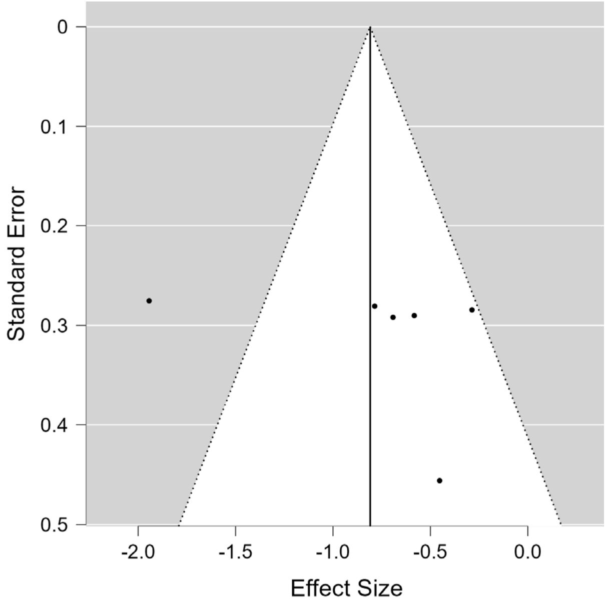

Processing Speed:

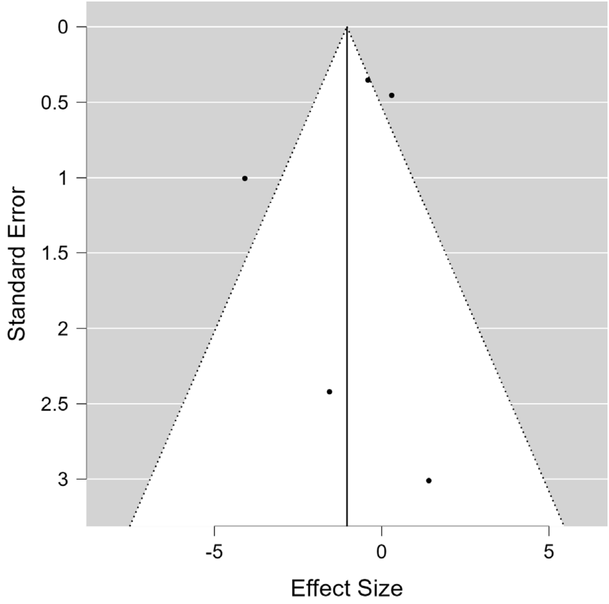

GAF:

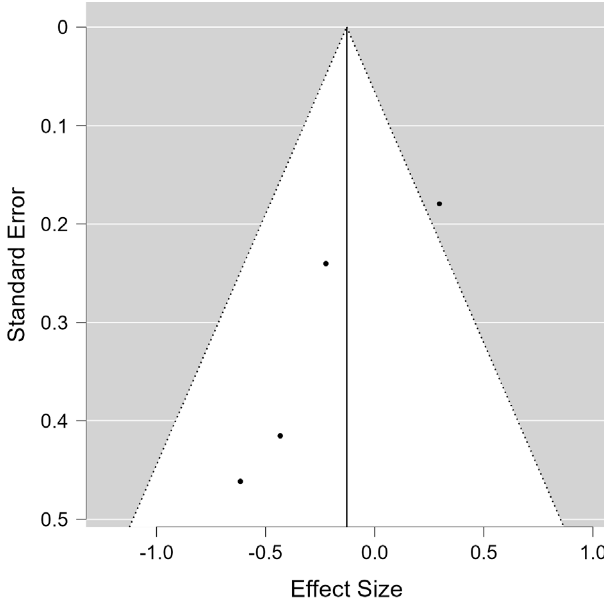

WM:

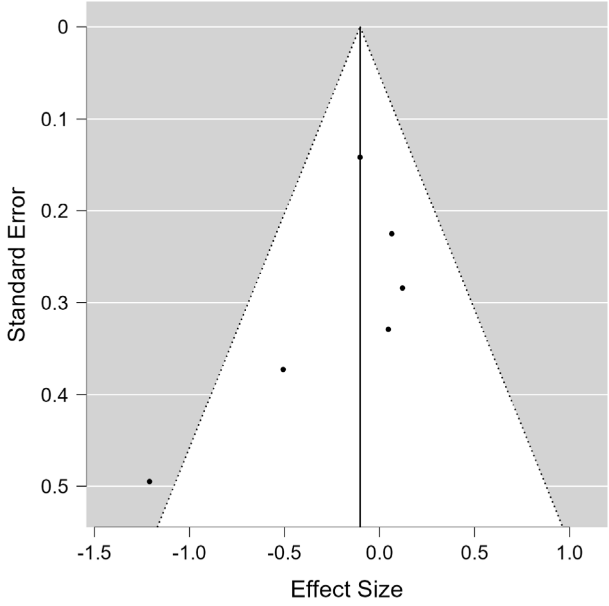

Executive Function:

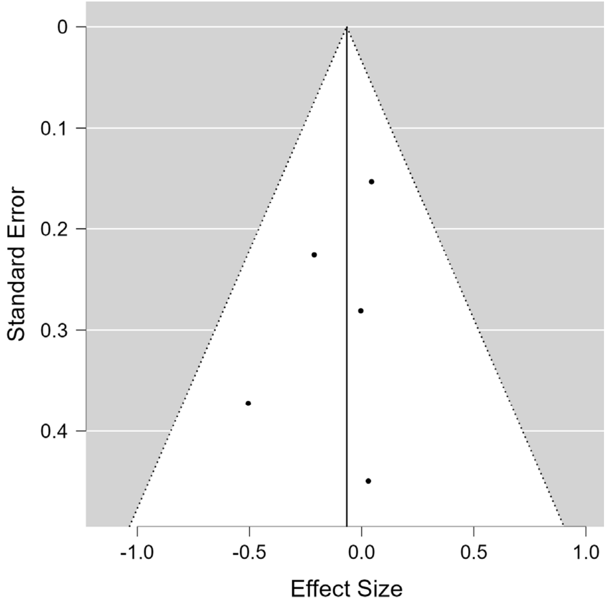

Problem Solving:

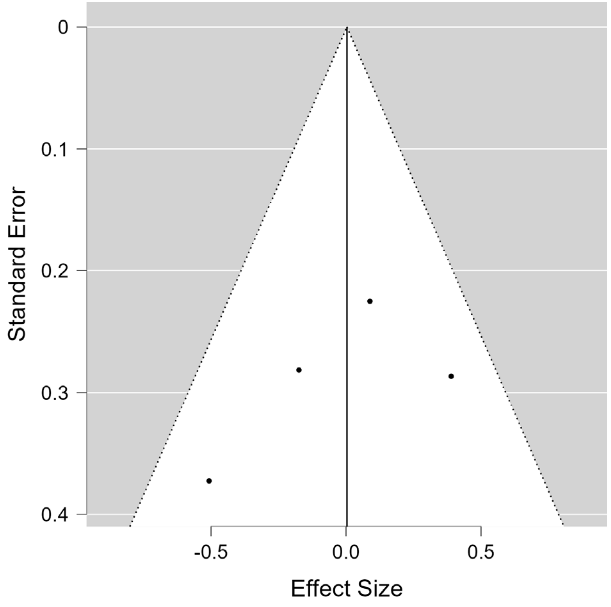

Verbal Learning:

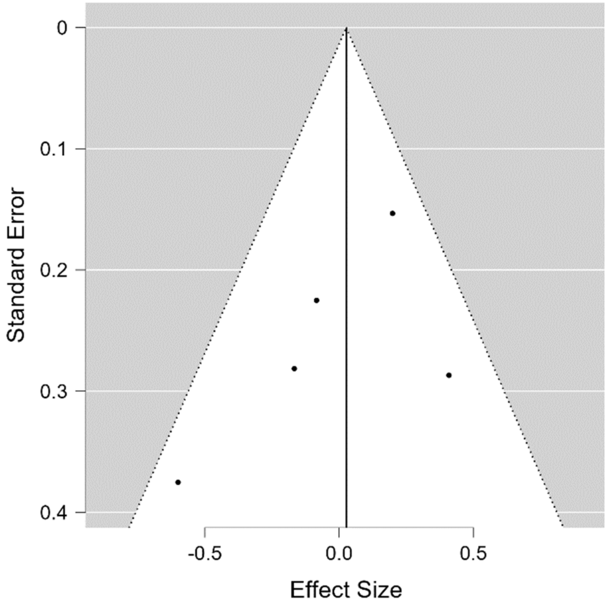

Funnel Plots Stroke

NIHSS

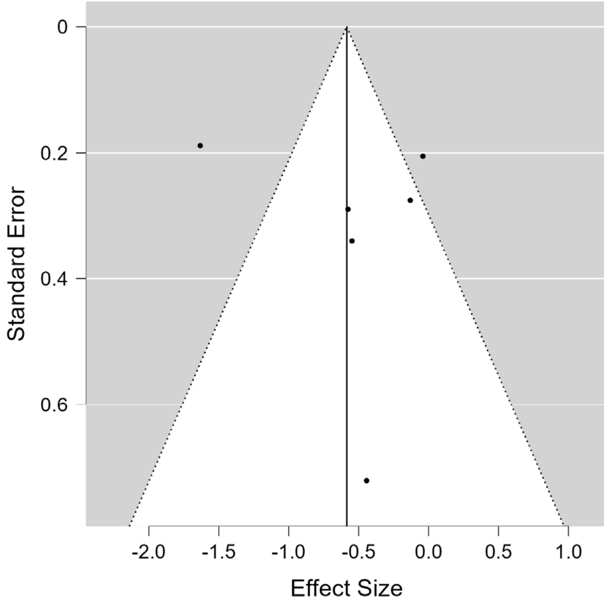

mRS Continuous:

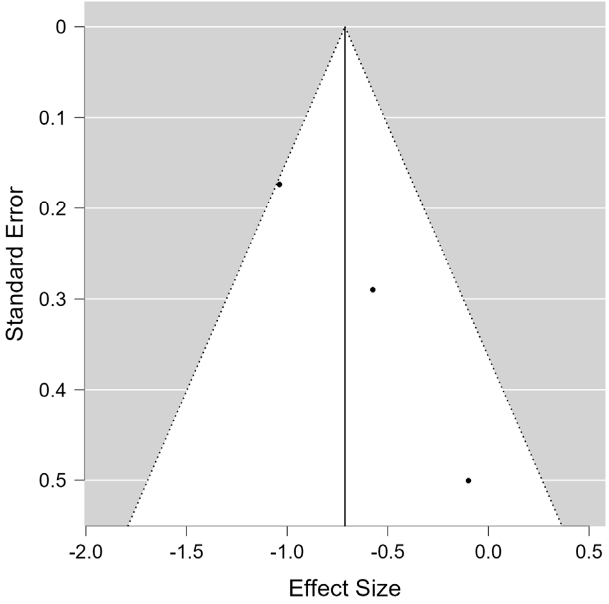

Bartel:

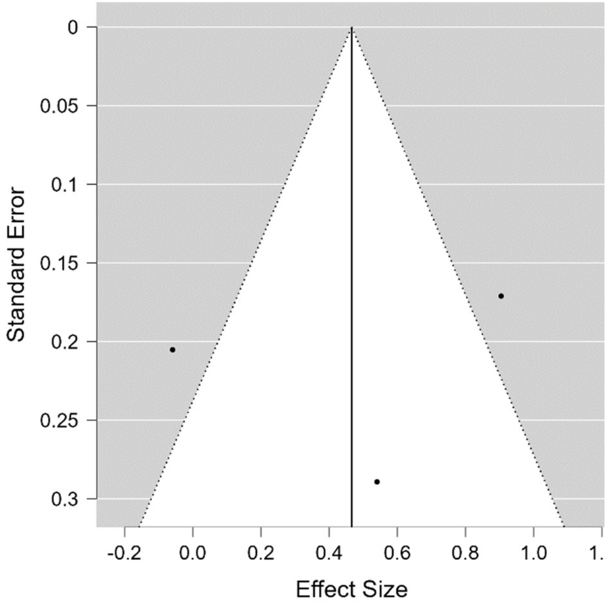

mRS Category change:

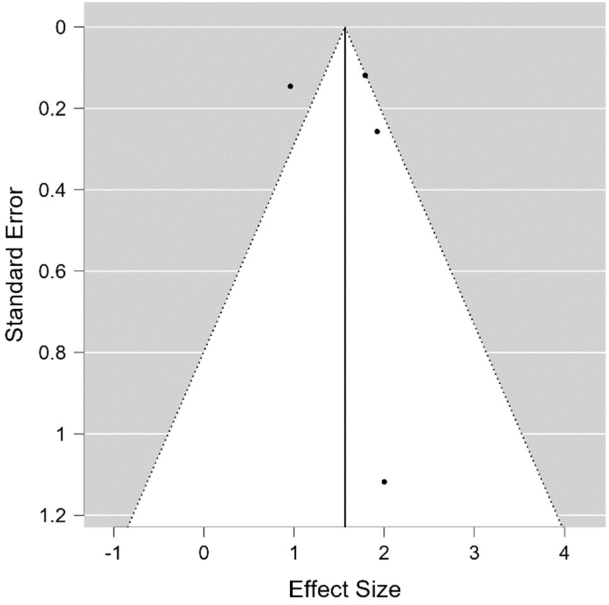

Supplement: Supplementary file 1 [file ijms-24-05250-s001.zip › ijms-2237842-supplementary.pdf]
